# Supplementary material for: Sub-3 Å Cryo-EM Structures of Necrosis Virus Particles via the Use of Multipurpose TEM with Electron Counting Camera
Source: Int J Mol Sci. 2021 Jun 25;22(13):6859. doi: 10.3390/ijms22136859 (PMC8268952; doi:10.3390/ijms22136859)
Supplement: Supplementary file 1 [file ijms-22-06859-s001.zip › ijms-1225291-supplementary.pdf]

Supplementary Materials

for

Sub-3 Å cryo-EM structures of necrosis virus particles via the use of multi-purposed TEM with electron counting camera

Chun-Hsiung Wang, Dong-Hua Chen, Shih-Hsin Huang, Yi-Min Wu, Yi-Yun Chen, Yeukuang Hwu, David Bushnell, Roger Kornberg and Wei-Hau Chang*

*Corresponding authors: weihau@gate.sinica.edu.tw (W.-H.C.)

This file includes:

Table S1 to S4
Figure S1 to S9

Table S1 Cryo-EM data collection summary

	Setting A	Setting A	Setting B	Setting C
Cryo-EM sample	DGNNV-WT	DGNNV-H94A	DGNNV-WT	MrNV
Cryo-EM equipment	JEM 2100F	JEM 2100F	FEI F-20	FEI Talos
Voltage (kV)	200	200	200	200
Cs (mm)	3.3	3.3	2.3	2.7
C2 aperture (um)	100	100	50	50
Spot size	2	2	6	3
Holder type	Side entry	Side entry	Side entry	Autoloader
Detector	DE-20	DE-20	K2	Falcon 3EC
Pixel array	5120 x 3840	5120 x 3840	3838 x 3710	4096 x 4096
Physical pixel size (um)	6.4	6.4	5.0	14.0
Operation mode	linear	linear	Counting (Super resolution)	linear
Data collection setting				
Magnification (nominal)	50,000	50,000	29,000	120,000
Pixel size (Å)	1.16	1.16	0.62	0.86
Defocus setting (μm)	-0.5 ~ -3.0	-0.5 ~ -3.0	-0.5 ~ -3.0	-1.0 ~ -2.5
Electron exposure (e^-/Å ^{2})	~ 40	~ 40	~ 50	~ 37
Exposure time (s)	2	2	10	3
Dose rate (e^-/Å ^{2} per second)	~ 20	~ 20	~ 5	~12
Total Frames (no.)	50	50	50	30

Table S2 Cryo-EM 3D refinement statistics (Processed with classical algorithms)

Dataset	Setting A (2100F-DE20) (DGNNV-WT)	Setting A (2100F-DE20) (DGNNV-H94A)	Setting B (F20-K2) (DGNNV-WT)	Setting C (Talos-Falcon3 EC) (MrNV)
Software	Auto3dem	Auto3dem	Auto3dem	cryoSPARC v1 & Relion 2
Selected frames (no.)	18	18	12	19
Dose rate (e^{-}/Å ^{2} per frame)	~ 0.8	~ 0.8	~ 1.0	~ 1.2
Electron exposure (e^{-}/Å ^{2})	~ 14	~ 14	~ 12	~ 23
Dose weighting	No	No	No	No
Defocus estimation (μm)	-0.56 ~ -2.78	-0.58 ~ -3.01	-0.39 ~ -2.91	-0.45 ~ -3.37
Micrographs stacks (no.)	588	517	1,009	1,353
Final particle images (no.)	41,318	15,182	29,575	19,049
Symmetry imposed	I (532)	I (532)	I (532)	I (I 1)
Pixel size (Å /pixel)	1.16	1.16	0.62 (bin1)<br>1.24 (bin2)	0.86
Nyquist limit (Å)	2.32	2.32	1.24 (bin1)<br>2.48 (bin2)	1.72
Map resolution (Å)[1]	3.56	3.78	2.86 (bin1)<br>3.18 (bin2)	2.92

[1]According to FSC=0.143.

Table S3 Cryo-EM 3D refinement statistics (Re-processed with modern algorithms)

Dataset	Setting A (2100F-DE20) (DGNNV-WT)	Setting A (2100F-DE20) (DGNNV-H94A)	Setting B (F20-K2) (DGNNV-WT)	Setting C (Talos-Falcon3 EC) (MrNV)
Software	Relion v3 & cryoSPARC v2	Relion v3 & cryoSPARC v2	Relion v3 & cryoSPARC v2	Relion v3 & cryoSPARC v2
Selected frames (no.)	50	50	50	30
Dose rate (e^-/Å^2 per frame)	~ 0.8	~ 0.8	~ 1.0	~ 1.2
Electron exposure (e^-/Å^2)	~ 40	~ 40	~ 50	~ 37
Dose weighting	Yes	Yes	Yes	Yes
Defocus estimation (μm)	-0.56 ~ -2.78	-0.61 ~ -3.06	-0.40 ~ -2.92	-0.40 ~ -3.44
Micrographs stacks (no.)	588	517	1,009	1,353
Final particle images (no.)	46,796	29,575	41,152	27,733
Symmetry imposed	I	I	I	I
Pixel size (Å /pixel)	1.16	1.16	1.24 (bin2)	0.86
Nyquist limit (Å)	2.32	2.32	2.48	1.72
Map resolution (Å)[1]	3.24	3.15	2.72	2.70

[1]According to FSC=0.143.

Table S4 Atomic model refinement statistics and validation scores for cryo-EM 3D reconstruction (Re-processed with modern algorithms)

Dataset	Setting A (2100F-DE20) (DGNNV-WT)	Setting A (2100F-DE20) (DGNNV-H94A)	Setting B (F20-K2) (DGNNV-WT)	Setting C (Talos-Falcon3 EC) (MrNV)
Software	Coot & Phenix	Coot & Phenix	Coot & Phenix	Coot & Phenix
Number of protein residues #	511	511	511	602
Number of metal ion #	Ca: 3	Ca: 3	Ca: 3	Ca: 7
Number of atoms #	3,910	3,895	3,910	4,753
Map CC (around atoms)	0.78	0.83	0.85	0.91
RMSD bond lengths (Å)	0.006	0.006	0.008	0.006
RMSD bond angles (°)	0.649	0.690	0.745	0.703
Clash score	7.19	6.31	8.47	6.96
Ramachandran favored (%)	97.03	97.82	98.42	95.3
Ramachandran allowed (%)	2.97	2.18	1.58	4.70
Ramachandran outliers (%)	0	0	0	0
Rotamer outliers (%)	0	0	0	0
C_{β} deviations	0	0	0	0
MolProbity score	1.57	1.39	1.46	1.71
EMRinger score	2.71	3.17	2.39	5.20

†According to FSC=0.143; # Statistics are given for one icosahedral asymmetric unit

Fig. S1

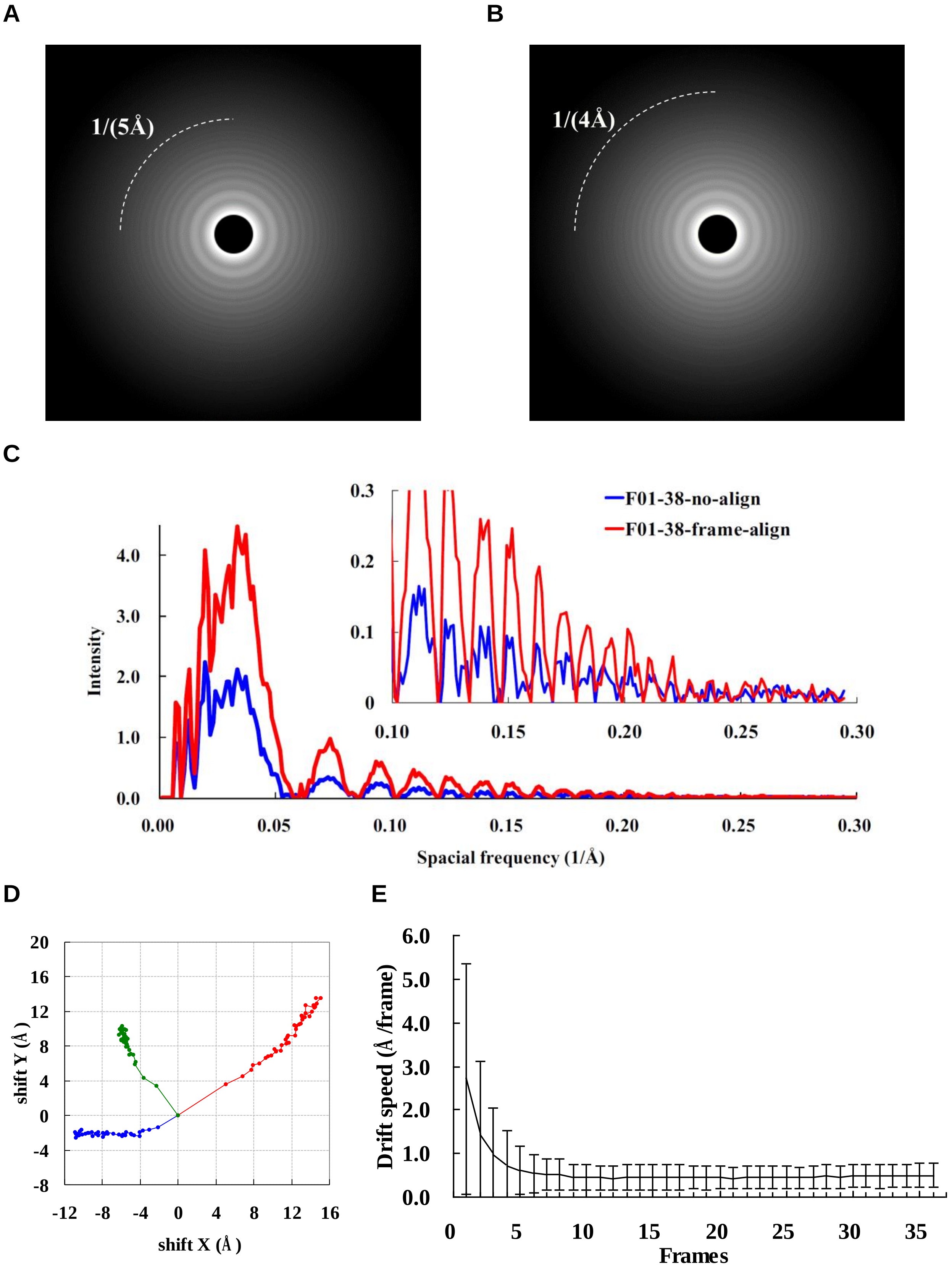

Fig. S1. Motion correction of 200 kV cryo-EM images of DGNNV on a direct electron camera. **(A)** 2D power spectra of DGNNV cryo-image before and **(B)** after move frame alignment. **(C)** 1D power spectra of DGNNV cryo-image after move frame alignment. The enlarged zoom of the high-resolution range is shown in the inset. The information limit in the accumulated image was extended from ~5 Å to ~4 Å . **(D)** Image movement paths of three micrographs. **(E)** Average drift speed kinetics measured by frame displacements from a total of 588 micrographs.

Fig. S2

A

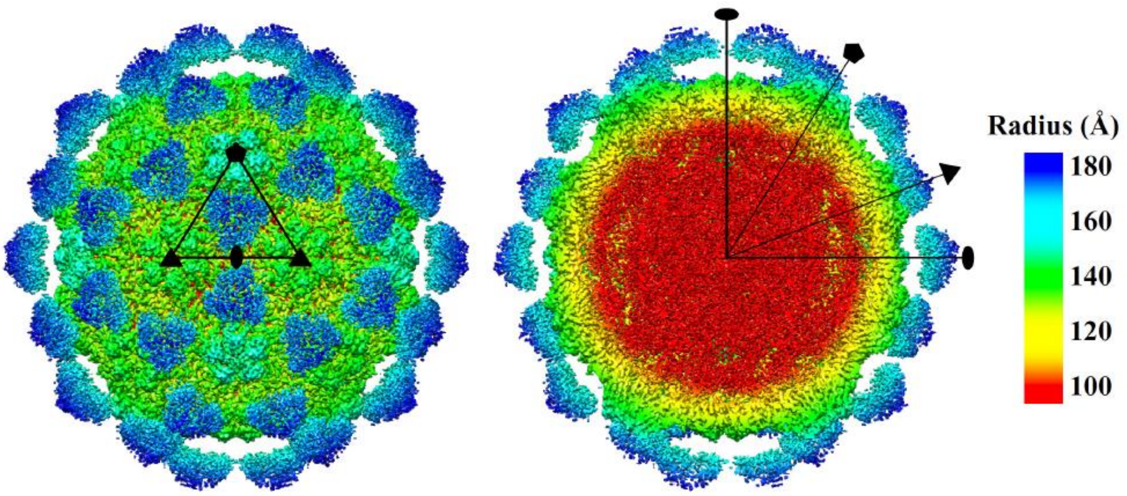

B

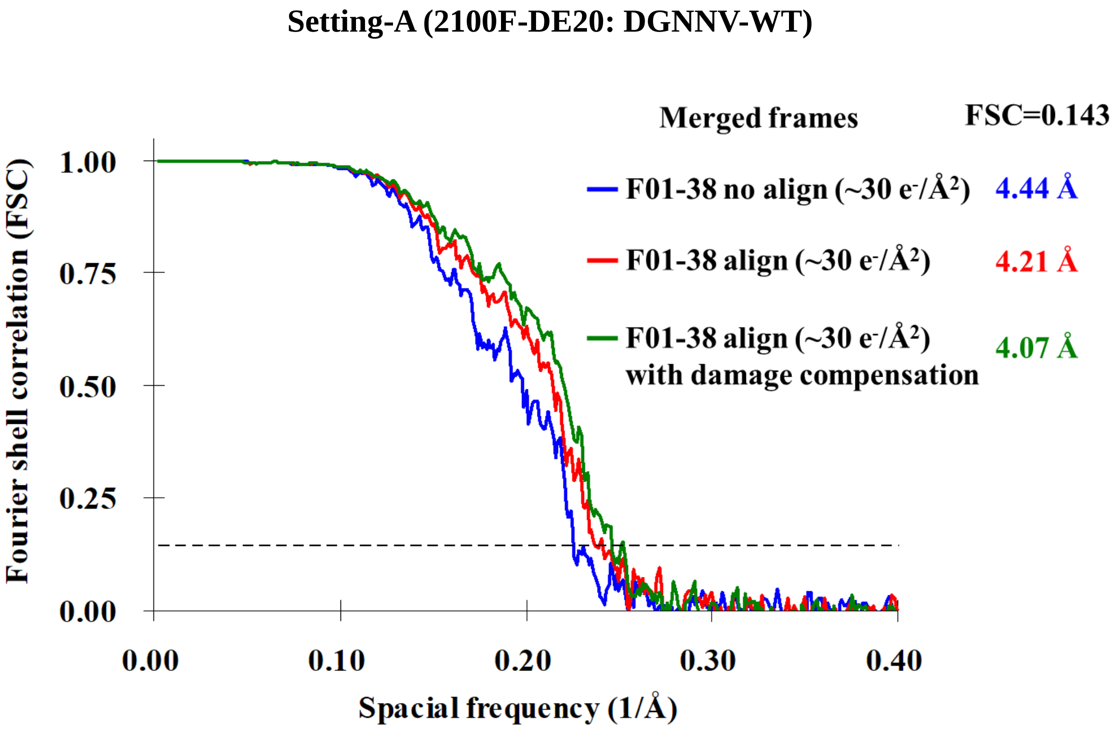

Fig. S2. Cryo-EM structure and resolution estimation of DGNNV particle from setting A. (A) Overall cryo-EM structure of DGNNV virus-like particle and the cutaway view to visualize the inside. The color indicates the distance from the center. **(B)** The resolution estimation of DGNNV cryo-EM reconstruction with imaging setting A with DE20 camera. The resolution was 4.21 Å for the drift-corrected reconstruction and 4.44 Å for the un-corrected reconstruction. With the drift-correction and radiation damage compensation strategy, the resolution of the reconstruction was improved to 4.07 Å.

Fig. S3

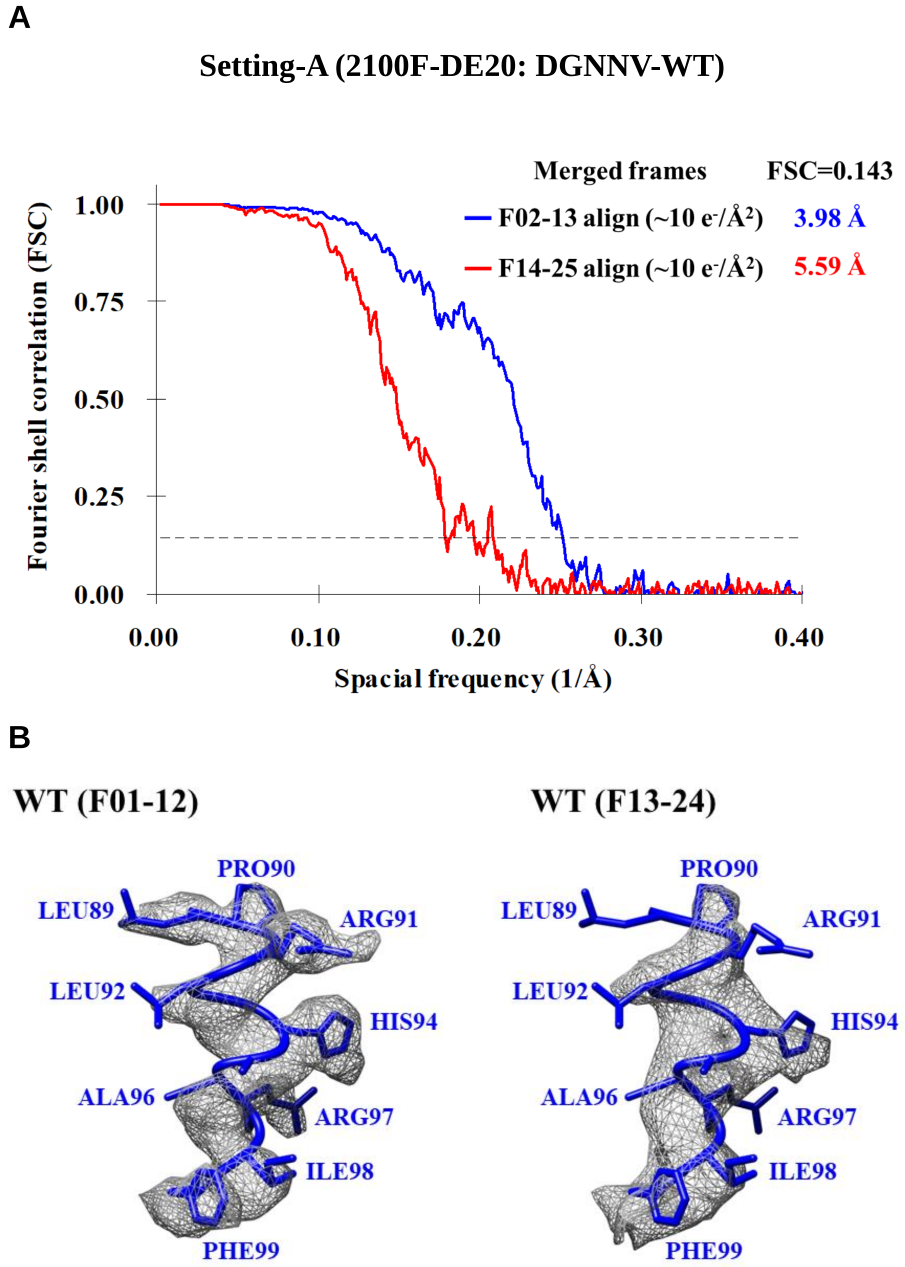

Fig. S3. Time dependence of the density map and resolution and dose effect on resolution. (A) Fourier shell correlation (FSC) analysis of two equal dose reconstructions: early (running average from 12 frames: 2th-13th) and late time (running average from 12 frames: 14th-25th). The resolutions of the maps were determined with the gold standard FSC at a cutoff of 0.143, indicated by horizon dash lines. **(B)** The density map of a α-helix in early time (*left panel*) and in late time (*right panel*) during which the side-chain densities vanished or reduced.

Fig. S4

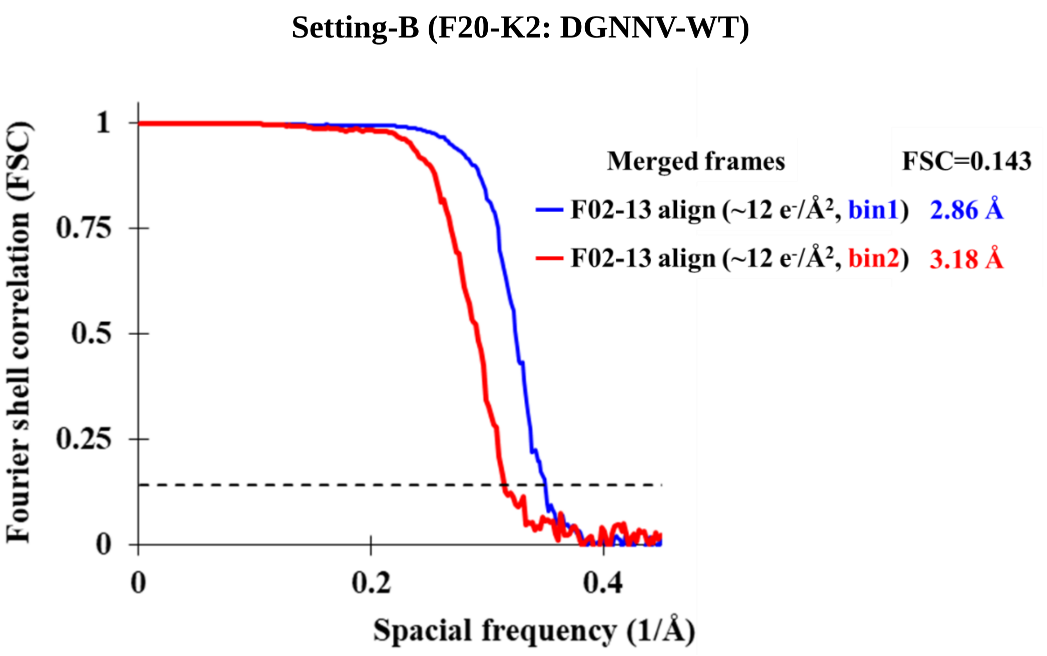

Fig. S4. The resolution estimation of the maps with imaging setting B with K2 camera. The resolution was 3.18 Å for the first binning of the data (bin2). By using the orientation parameters obtained from the binned data to refine the unbinned data (bin1), the resolution was slightly improved to 2.86Å. The resolutions were determined by the gold standard Fourier shell correlation (FSC) at a cutoff of 0.143. Horizontal dashed line: FSC = 0.143.

Fig. S5.

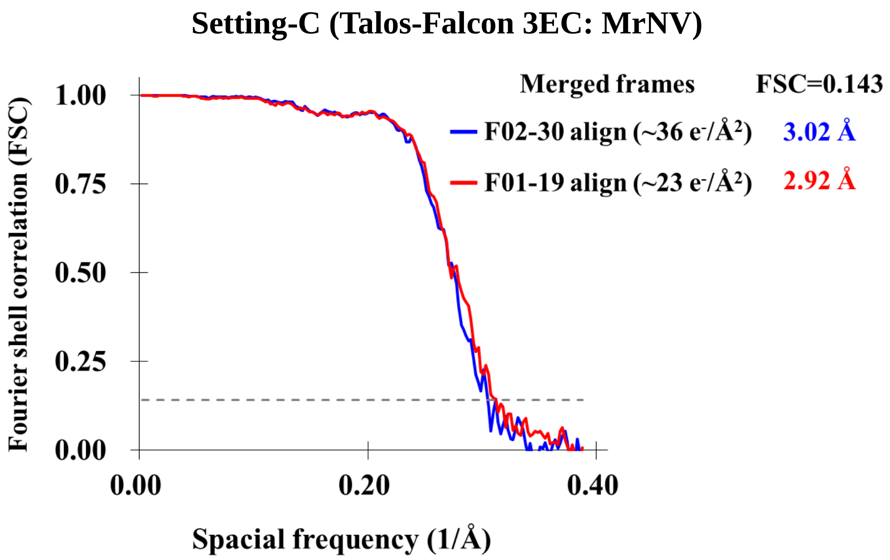

Fig. S5. Resolution estimation of freshwater prawn *Macrobrachium resenbergii* VLPs. The resolution estimation of MrNV VLPs was determined by gold-standard Fourier shell correlation (FSC) at a cutoff of 0.143 (horizontal line).

Fig. S6.

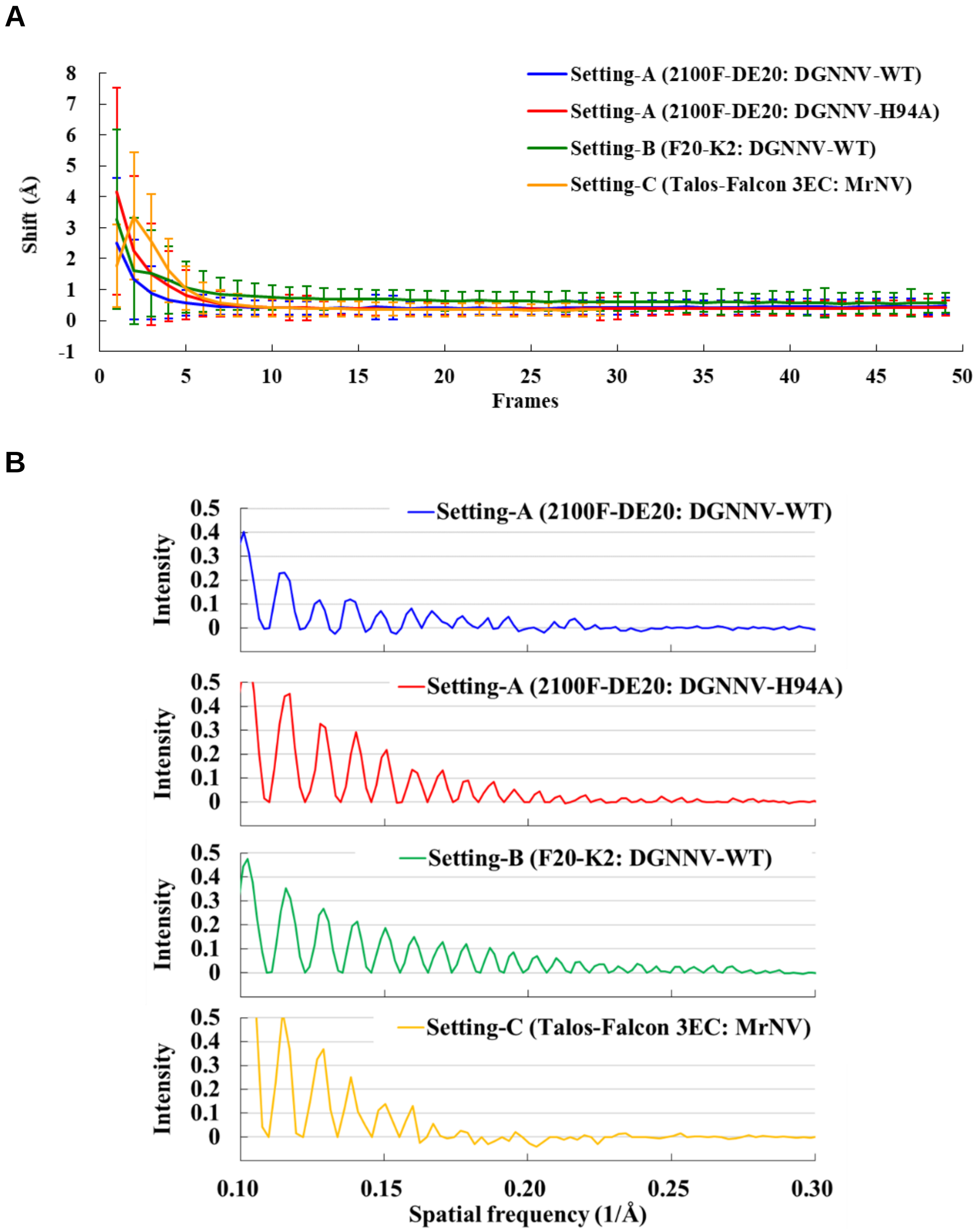

Fig. S6. Comparison of the average motion and power spectra among different cryo-EM settings. (A) Average drift speed kinetics measured by frame displacements from all micrographs among different cryo-EM settings. **(B)** 1D power spectra of cryo-image after move frame alignment.

Fig. S7.

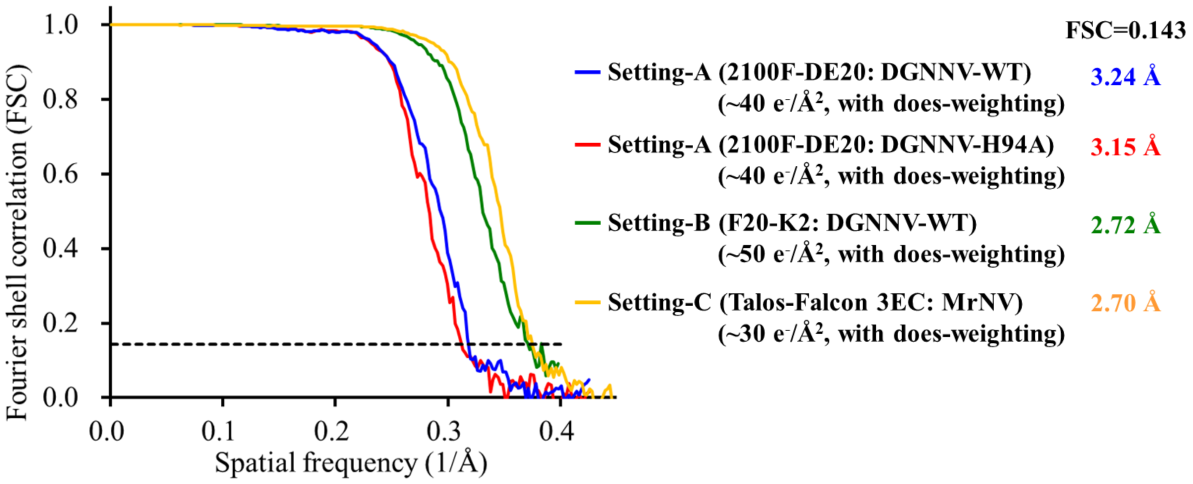

Fig. S7. Resolution estimation of DGNNV and MrNV VLPs cryo-EM reconstruction with modern algorithm. The resolution was 3.24 Å for DGNNV WT-VLP and 3.15 Å for DGNNV H94A-VLP with setting A (2100F-DE20). The resolution was 2.72 Å for DGNNV WT-VLP with setting B (F20-K2) and 2.70 Å for MrNV DN30-VLP with setting C (Talos-Falcon 3EC). The resolution estimation was determined by gold-standard Fourier shell correlation (FSC) at a cutoff of 0.143 (horizontal line).

Fig. S8.

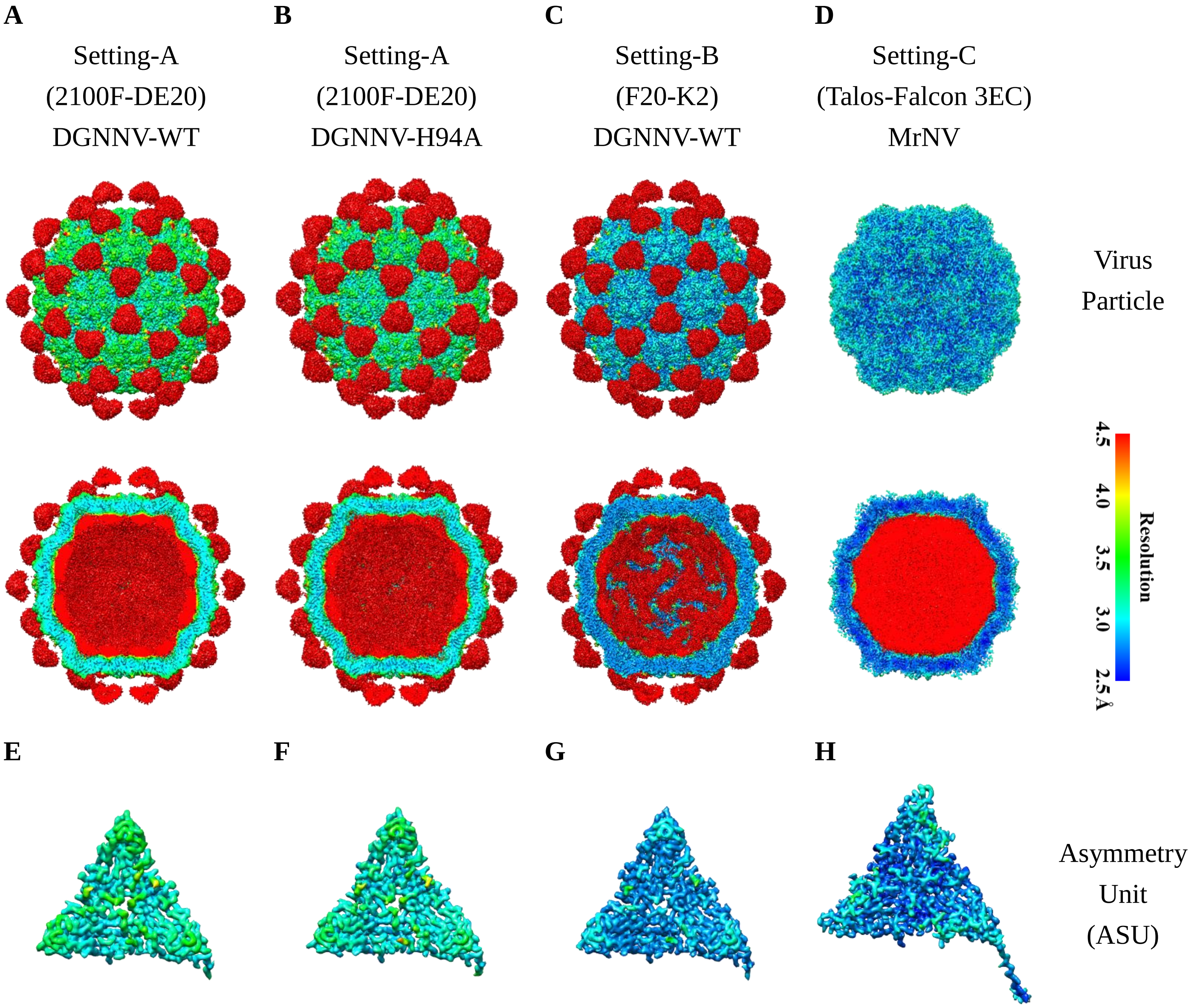

Fig. S8 Local resolution estimation of cryo-EM structures reconstructed by modern algorithm. (A) Local resolutions estimation of cryo-EM reconstruction of DGNNV WT-VLP, **(B)** DGNNV H94A-VLP with setting A (2100F-DE20), **(C)** DGNNV WT-VLP with setting B (F20-K2), and **(D)** MrNV DN30-VLP with setting C (Talos-Falcon 3EC). The cutaway view to visualize the inside. **(E)** Local resolution estimation of a shell capsid asymmetric unit of DGNNV WT-VLP, **(F)** DGNNV H94A-VLP with setting A (2100F-DE20), **(G)** DGNNV WT-VLP with setting B (F20-K2), and **(H)** MrNV DN30-VLP with setting C (Talos-Falcon 3EC). The maps were colored by local resolution reported by cryoSPARC.

Fig. S9.

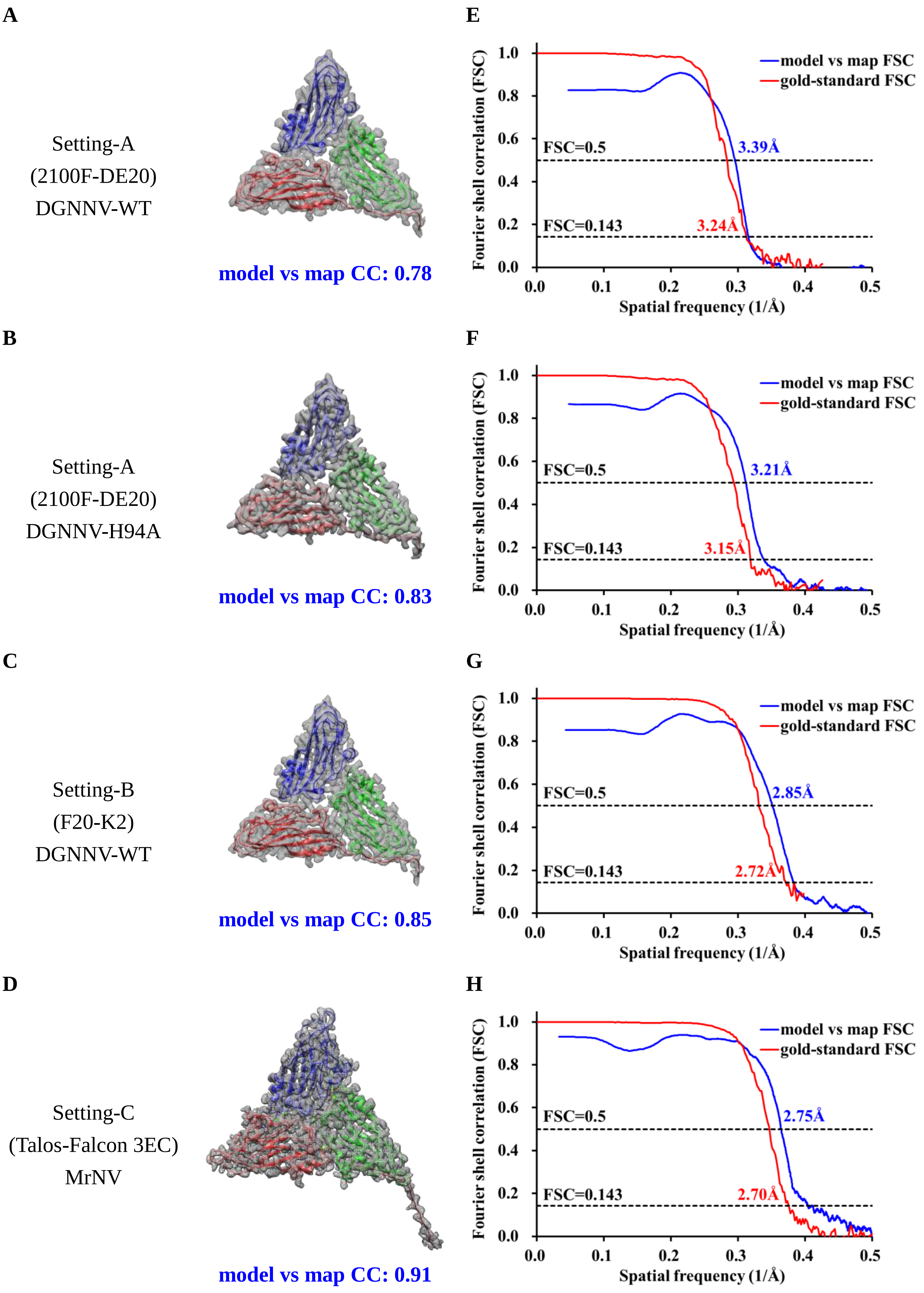
A
Setting-A
(2100F-DE20)
DGNNV-WT
model vs map CC: 0.78
B
Setting-A
(2100F-DE20)
DGNNV-H94A
model vs map CC: 0.83
C
Setting-B
(F20-K2)
DGNNV-WT
model vs map CC: 0.85
D
Setting-C
(Talos-Falcon 3EC)
MrNV
model vs map CC: 0.91
E
model vs map FSC
gold-standard FSC
FSC=0.5
FSC=0.143
3.39Å
3.24Å
Fourier shell correlation (FSC)
Spatial frequency (1/Å)
F
model vs map FSC
gold-standard FSC
FSC=0.5
FSC=0.143
3.21Å
3.15Å
Fourier shell correlation (FSC)
Spatial frequency (1/Å)
G
model vs map FSC
gold-standard FSC
FSC=0.5
FSC=0.143
2.85Å
2.72Å
Fourier shell correlation (FSC)
Spatial frequency (1/Å)
H
model vs map FSC
gold-standard FSC
FSC=0.5
FSC=0.143
2.75Å
2.70Å
Fourier shell correlation (FSC)
Spatial frequency (1/Å)

Fig. S9. The atomic model building and the validation of cryo-EM structure models. (A) The atomic model building of a shell capsid asymmetric unit of DGNNV WT-VLP, **(B)** DGNNV H94A-VLP with setting A (2100F-DE20), **(C)** DGNNV WT-VLP with setting B (F20-K2), and **(D)** MrNV DN30-VLP with setting C (Talos-Falcon 3EC). The cross correlated value (CC) between the corresponding model and map was indicated. **(E)** Validation of cryo-EM structural model of DGNNV WT-VLP, **(F)** DGNNV H94A-VLP with setting A (2100F-DE20), **(G)** DGNNV WT-VLP with setting B (F20-K2), and **(H)** MrNV DN30-VLP with setting C (Talos-Falcon 3EC). Model-map FSC curves of DGNNV and MrNV cryo-EM maps were generated from MolProbity. FSC curves between the atomic model and the cryo-EM map with indicated resolution at FSC = 0.5 are colored in blue. Gold-standard FSC curves between the two half maps with indicated resolution at FSC = 0.143 are colored in red.
